# Functional MRI Motor Imagery Tasks to Detect Command Following in Traumatic Disorders of Consciousness

**DOI:** 10.3389/fneur.2017.00688

**Published:** 2017-12-18

**Authors:** Yelena G. Bodien, Joseph T. Giacino, Brian L. Edlow

**Affiliations:** ^1^Center for Neurotechnology and Neurorecovery, and Laboratory for NeuroImaging of Coma and Consciousness, Department of Neurology, Massachusetts General Hospital, Harvard Medical School, Boston, MA, United States; ^2^Department of Physical Medicine and Rehabilitation, Spaulding Rehabilitation Hospital, Harvard Medical School, Boston, MA, United States; ^3^Department of Psychiatry, Massachusetts General Hospital and Harvard Medical School, Boston, MA, United States; ^4^Athinoula A. Martinos Center for Biomedical Imaging, Massachusetts General Hospital and Harvard Medical School, Charlestown, MA, United States

**Keywords:** traumatic brain injury, consciousness, awareness, functional magnetic resonance imaging, motor imagery

## Abstract

Severe traumatic brain injury impairs arousal and awareness, the two components of consciousness. Accurate diagnosis of a patient’s level of consciousness is critical for determining treatment goals, access to rehabilitative services, and prognosis. The bedside behavioral examination, the current clinical standard for diagnosis of disorders of consciousness, is prone to misdiagnosis, a finding that has led to the development of advanced neuroimaging techniques aimed at detection of conscious awareness. Although a variety of paradigms have been used in functional magnetic resonance imaging (fMRI) to reveal covert consciousness, the relative accuracy of these paradigms in the patient population is unknown. Here, we compare the rate of covert consciousness detection by hand squeezing and tennis playing motor imagery paradigms in 10 patients with traumatic disorders of consciousness [six male, six acute, mean ± SD age = 27.9 ± 9.1 years, one coma, four unresponsive wakefulness syndrome, two minimally conscious without language function, and three minimally conscious with language function, per bedside examination with the Coma Recovery Scale-Revised (CRS-R)]. We also tested the same paradigms in 10 healthy subjects (nine male, mean ± SD age = 28.5 ± 9.4 years). In healthy subjects, the hand squeezing paradigm detected covert command following in 7/10 and the tennis playing paradigm in 9/10 subjects. In patients who followed commands on the CRS-R, the hand squeezing paradigm detected covert command following in 2/3 and the tennis playing paradigm in 0/3 subjects. In patients who did not follow commands on the CRS-R, the hand squeezing paradigm detected command following in 1/7 and the tennis playing paradigm in 2/7 subjects. The sensitivity, specificity, and accuracy (ACC) of detecting covert command following in patients who demonstrated this behavior on the CRS-R was 66.7, 85.7, and 80% for the hand squeezing paradigm and 0, 71.4, and 50% for the tennis playing paradigm, respectively. Overall, the tennis paradigm performed better than the hand squeezing paradigm in healthy subjects, but in patients, the hand squeezing paradigm detected command following with greater ACC. These findings indicate that current fMRI motor imagery paradigms frequently fail to detect command following and highlight the need for paradigm optimization to improve the accuracy of covert consciousness detection.

## Introduction

Patients with severe traumatic brain injury (TBI) experience a period of impaired consciousness characterized by disturbances in arousal and awareness. This disorder of consciousness (DoC) may resolve acutely [i.e., in the intensive care unit (ICU)] or may be prolonged, extending weeks, months, or even years post-injury (1). The spectrum of behavioral states that comprise DoC includes coma, unresponsive wakefulness syndrome (UWS, also known as vegetative state) (2, 3), minimally conscious state (MCS) (4), and post-traumatic confusional state (5, 6). Accurate assessment of a patient’s level of consciousness (LoC) is critically important to prognosis, as patients who have recovered consciousness (i.e., MCS) and especially language function have a higher likelihood of regaining cognitive function than those who have not (i.e., coma and UWS) (7–10). Thus, assessment of LoC drives early decisions about aggressive treatment and access to rehabilitative care. An inaccurate diagnosis may also prevent autonomous decision-making in patients who retain the capacity to do so.

Despite the critical importance of accurately defining a patient’s LoC, the current standard for assessment in this patient population is bedside clinical examination, a method that is prone to inaccuracies due to patient impairments (e.g., speech and motor deficits that prevent verbalization or movement to command) and examiner bias. The approximate rate of misdiagnosing a conscious patient as unconscious is 40% (11–14). Standardized behavioral tools, such as the Coma Recovery Scale-Revised (CRS-R) (15), have helped improve the accuracy and precision of the bedside assessment, but the behavioral diagnosis is potentially susceptible to misinterpretation of subtle and inconsistent behaviors. Recently published guidance on the optimal frequency of CRS-R assessment may further improve the accuracy of behavioral assessment (16), but even frequent assessments with the CRS-R may fail to detect consciousness in persons whose capacity for volitional brain function is masked by limitations in self-expression. Objective markers of consciousness are therefore needed to ensure accurate diagnosis and to guide care management.

To circumvent some of the limitations of the bedside behavioral examination, recent studies have attempted to elicit evidence of consciousness by asking a patient to perform a mental imagery task in a magnetic resonance imaging (MRI) scanner (17–24). These functional MRI (fMRI) motor imagery tasks are not confounded by speech or motor impairment and therefore may provide additional information about a patient’s LoC that cannot be obtained by a behavioral assessment. Moreover, the magnitude, signal characteristics, and neuroanatomic location of brain activations detected by fMRI can be analyzed using predetermined objective algorithms that are independent of observer bias or variations in the administration and scoring of standardized behavioral measures. Several fMRI studies have identified persons with acute (24) and chronic (21, 25) DoC who demonstrate cognitive-motor dissociation (CMD) (26), which is defined by fMRI evidence of command following in the absence of behavioral evidence of command following.

Currently, there is a lack of consensus regarding which fMRI paradigms are best suited to elicit covert command following and hence a diagnosis of CMD. Although early studies used covert object naming (21), and some have employed covert counting of target words (27), most recent fMRI investigations have focused on spatial navigation and motor imagery tasks (e.g., imagine playing tennis, swimming, or squeezing the right or left hand) (17). For a review of tasks used to elicit command following in patients with DoC see Rossetti and Laureys (28) and Laureys and Schiff (29). In 2007, Boly and colleagues (30) compared the robustness of brain activation to four task-based fMRI imagery paradigms in healthy subjects: spatial navigation (imagine walking around the rooms of a house), auditory imagery (imagine a familiar song), motor imagery (imagine hitting a tennis ball), and visual imagery (imagine familiar faces). They found that the spatial navigation and tennis imagery tasks provided the most robust results in healthy subjects. Consequently, tennis motor imagery has been utilized frequently over the past decade to identify CMD in patients with DoC.

Although the fMRI tennis imagery task seems to be a viable complement to the bedside examination of patients with DoC, several studies have found high false-negative rates (FNRs) using this task (i.e., patient and healthy subjects who have behavioral evidence of command following do not demonstrate the expected activations on tennis imagery fMRI tasks) (17, 31). A hand squeezing motor imagery task has been used successfully in EEG studies (32, 33) and may be a more robust paradigm for use in the ICU, as it parallels the clinical bedside examination and may be less cognitively burdensome than imagining playing tennis. It remains unknown whether tennis playing imagery or hand squeezing imagery is a more effective paradigm for detecting conscious awareness.

Our aim in the present study was to compare fMRI activation in response to a tennis playing and hand squeezing paradigm in patients with traumatic DoC. The hand squeezing paradigm was chosen because it is a simple motor response that closely resembles the bedside behavioral examination, which often includes a “squeeze my hand” instruction to elicit command following. In addition, hand squeezing is more universal than playing tennis, which may be imagined differently and with varying intensity depending on an individual’s exposure to the sport. The hand squeezing task has been used successfully in other studies in acute and chronic DoC (24, 32, 33). We hypothesized that hand squeezing motor imagery will be detected with greater frequency than tennis playing motor imagery in patients diagnosed with acute and chronic traumatic DoC and in healthy controls.

## Materials and Methods

### Experimental Design

This study was carried out in accordance with a protocol approved by the Partners Institutional Review Board. Patient surrogate decision-makers gave written informed consent in accordance with the Declaration of Helsinki. The patient cohort was prospectively recruited from an ICU, an outpatient follow-up neurology clinic, and an affiliated long-term acute hospital. Inclusion criteria were as follows: (1) age 18–65 years; and (2) head trauma with Glasgow Coma Scale score of 3–8 with no eye opening for at least 24 h after injury. Exclusion criteria were as follows: (1) life expectancy less than 6 months, as determined by a treating clinician; (2) prior severe brain injury or neurodegenerative disease; (3) penetrating TBI with intracranial metal or other body metal precluding MRI; and (4) no fluency in English prior to the injury (because the paradigms were administered in English).

Surrogate decision-makers were approached for consent ≥ 24 h after injury. For the ICU cohort, fMRI was performed as soon as the patient was clinically stable for transport to the MRI scanner, as determined by the treating ICU physicians and nurses. Patients with chronic DoC were scanned when they returned to the hospital for an outpatient clinic appointment or an inpatient hospitalization (e.g., for cranioplasty). Administration of sedative, anxiolytic, and/or analgesic medications was permitted for patient safety or comfort, at the discretion of the treating clinicians.

Age-matched healthy subjects were enrolled as a comparison group. Healthy subjects had no history of neurological, psychiatric, cardiovascular, pulmonary, renal, or endocrinological disease. They provided written informed consent and underwent the same fMRI protocols as the patients. All patient and healthy subject MRI scans were performed on the same scanner.

### Neurobehavioral Assessment

Demographic and clinical data were collected at the time of enrollment in accordance with the National Institutes of Health Common Data Element Guidelines for TBI.^1^ LoC was characterized *via* behavioral evaluation with the CRS-R or based on criteria derived from the Confusion Assessment Protocol immediately prior to the fMRI (6, 34). Based on the CRS-R, each patient’s LoC was defined as coma (no arousal or awareness), UWS (return of arousal but no awareness of self or the environment) (2, 3), or MCS (return of arousal and reliable but inconsistent evidence of awareness) (4). MCS was further subdivided into MCS− and MCS+ with the distinguishing feature being the presence of language function (i.e., at least one of the following: command following, object-recognition, or intelligible verbalization) in patients diagnosed as MCS+ (35, 36). Emergence from MCS was marked by recovery of either functional use of two common objects or basic accurate communication. The neurobehavioral assessment was conducted immediately prior to the fMRI scan. All behavioral evaluations were conducted by Brian L. Edlow.

### MRI Data Acquisition

Magnetic resonance imaging data were acquired with a 32-channel head coil on a 3 T Siemens Skyra MRI scanner (Siemens Medical Solutions, Erlangen, Germany) located in the Neurosciences ICU. Auditory stimuli were presented *via* MRI-compatible earphones (Newmatic Medical, Caledonia, MI, USA) connected to the scanner’s sound system. The blood-oxygen level dependent (BOLD) fMRI sequence utilized the following parameters: echo time (TE) = 30 ms, repetition time (TR) = 4,000 ms, in-plane resolution = 2.0 mm × 2.0 mm, slice thickness = 2 mm, interslice gap = 2.5 mm, matrix = 94 × 94, field of view (FOV) = 192 mm × 192 mm, 49 slices, 2× GRAPPA acceleration. Image acquisition parameters differed for one subject (P10) due to a change in the fMRI protocol motivated by decreasing scan time. For this subject, the fMRI sequence TE was reduced to 25 ms and TR to 3,000 ms. High-spatial resolution 3D T1-weighted multi-echo magnetization prepared gradient echo (MEMPRAGE) anatomical images were acquired for registration purposes (37): FOV = 256 mm × 256 mm, acquisition matrix = 256 × 256, 176 sagittal slices (thickness 1 mm), 3× GRAPPA acceleration, TE = 1.69, 3.55, 5.41, and 7.27 ms, TR = 2,530 ms, inversion time = 1,200–1,300 ms, 1.0 mm^3^ isotropic resolution, and flip angle = 7°.

### fMRI Paradigms

Two fMRI motor imagery paradigms—hand squeezing and tennis playing—were performed as part of a larger fMRI and EEG study. fMRI data from the hand squeeze task have been previously reported for P1-5 and C1-10 (24). Each motor imagery fMRI paradigm utilized a block design and was comprised of two runs, with each run containing three 24-s rest blocks and two 24-s stimulation blocks. In total, 144 s of rest data and 96 s of stimulation data were analyzed for each paradigm. Prior to the first rest block, 36 s of data (9 s for P10) were acquired to obtain a stable baseline BOLD signal. These data were excluded from analysis.

The hand squeeze motor imagery task always preceded the tennis motor imagery task because the former paradigm was part of the primary aim of a larger study (24) and the latter paradigm was added as part of a secondary study aim after initiation of data collection. Similar to other studies in DoC (32, 33), subjects were instructed to imagine squeezing their right hand or to rest. During the task, instructions to “keep squeezing” or to “keep resting” were repeated at 6-s intervals. The tennis playing task was identical to the hand squeezing task except that subjects were instructed to imagine playing tennis or to rest. During the task, instructions of “keep playing” or “keep resting” were repeated at 6-s intervals. Instructions administered before and during the fMRI are detailed in Table S1 in Supplementary Material.

### fMRI Data Analysis

In a first-level analysis of the individual runs, fMRI data processing was performed using the FMRI Expert Analysis Tool (FEAT) version 6.00 in FSL 5.0.7 (FMRIB’s Software Library^2^). Motor imagery stimuli were contrasted against rest. *Z* statistic images were thresholded (*Z* > 3.1) and a corrected cluster significance threshold of *P* = 0.05 was used. Higher-level analysis was carried out using a fixed effects model (FLAME in FSL) (38, 39). The statistical threshold for cluster significance (*Z* > 3.1) and the size of the Gaussian kernel (FWHM 10 mm) were both selected to decrease false positive cluster activations (40). Additional details on analysis have been published elsewhere (24).

We used FEATQuery in FSL to quantify the percentage of voxels activated within a prespecified region of interest (ROI). For healthy subjects, we defined a positive response by the criterion that >0% of ROI voxels met the aforementioned statistical threshold. For patients, we defined a positive response by two criteria, consistent with a recently proposed definition (24): (1) >0% of ROI voxels met the statistical threshold; and (2) the percentage of activated ROI voxels was above the 2.5th percentile of a normal range (2.5th–97.5th percentile) derived from the healthy subjects’ data for each paradigm. This quantitative approach was completely automated and did not require subjective interpretation by clinical or research staff, thereby reducing potential bias that may have been introduced by knowledge of the patient’s behavioral diagnosis.

### fMRI Regions of Interest

**Figure 1:**
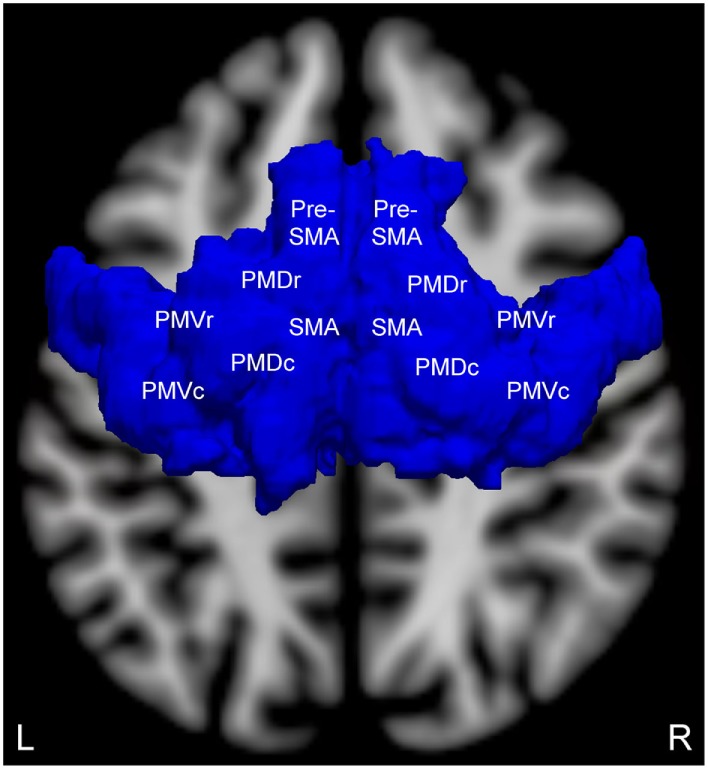
Brodmann area 6 region of interest (superior view) for measuring motor imagery functional magnetic resonance imaging (fMRI) activation. This region of interest contains the supplementary motor area (SMA), pre-SMA, and the four components of the bilateral premotor cortices: premotor dorsal rostral (PMDr), premotor dorsal caudal (PMDc), premotor ventral rostral (PMVr), and premotor ventral caudal (PMVc). Adapted and reproduced with permission from Edlow et al. (24).

We selected an *a priori* ROI based upon fMRI studies of motor imagery in patients with chronic traumatic DoC and healthy subjects, as well as a recently published study of patients with acute traumatic DoC that used this same ROI (24). The bilateral supplementary motor areas (SMA) from the Harvard-Oxford Cortical Structural Atlas and premotor cortices (PMC) from the Juelich Histological Atlas (41) were combined as a single ROI (Figure 1). This ROI was transformed from standard atlas space into patient native fMRI space for analysis, consistent with prior fMRI studies of patients with DoC (17, 20, 24, 42, 43).

### Statistical Analysis

Using the CRS-R as the reference standard and the motor imagery fMRI responses as the test criteria, we assessed the reliability of each paradigm in detecting behavioral evidence of command following by calculating the true-positive rate (TPR; i.e. sensitivity), true-negative rate (TNR; i.e. specificity), FNR and the false-positive rate (FPR) with 95% exact confidence interval (CI) in the patient cohort. Notably, the FPR may include both false positives (i.e., patients wrongly identified on fMRI as being able to follow commands) and cases of dissociation between behavioral and fMRI responses (i.e., CMD caused by speech or motor impairments or other confounding factors). The accuracy (ACC) of each fMRI paradigm for detecting command following was calculated as (TP + TN)/(TP + FP + TN + FN). We also calculated the TP proportions in the healthy subject cohort but not the other metrics because all healthy subjects exhibited behavioral evidence of command following.

We tested for an association between sedation (dichotomized as presence or absence) and fMRI responses, as well as between sedation and LoC at the time of fMRI (dichotomized as presence or absence of command following), using a 2 × 2 Fisher’s Exact Test. Statistical analyses were performed in STATA v14s (44).

## Results

### Demographics and Clinical Characteristics

Hand squeeze and tennis playing fMRI data were acquired in a convenience sample of 12 patients. One of these subjects was excluded due to severe artifact resulting from a ventriculoperitoneal shunt that prevented spatial normalization of the data. A second subject was excluded due to errors in the data acquisition of DICOM images. The final patient cohort included 10 subjects (six male, mean ± age = 27.9 ± 9.1, six acute, one coma, four UWS, two MCS−, and three MCS+). Demographic and clinical data are presented in Table 1. Ten age-matched healthy subjects were recruited (nine male, mean ± SD age = 28.5 ± 9.4, see Table S2 in Supplementary Material). There was no statistical difference in the proportion of males to females (chi-squared, *P* > 0.10) or in the age of the subjects (*t*-test, *P* > 0.88) between the patient and healthy subject groups.

**Table 1:** Patient demographics and clinical characteristics.

ID	Age (years)	Sex	TBI mechanism	iGCS	Day of fMRI	CRS-R at fMRI	CRS-R subscale scores at fMRI	LoC at fMRI
P1	27	F	Fall	3	8	1	A0V0M1O0C0Ar0	Coma
P2	18	M	Fall	3–7	4	12	A3V2M5O1C0Ar1	MCS+
P3	51	M	Ped vs. car	3	8	3	A0V0M1O1C0Ar1	UWS
P4	29	M	Ped vs. car	4–7	7	3	A0V0M3O0C0Ar0	MCS−
P5	33	M	Fall	3–4	3	12	A4V2M5O0C0Ar1	MCS+
P6	25	M	MVA	3–6 T	183	15	A4V3M3O2C1Ar2	MCS +
P7	22	F	Ped vs. car	3–3 T	162	5	A1V1M1O1C0Ar1	UWS
P8	26	F	Ped vs. truck	3–3 T	12	2	A0V0M1O0C0Ar1	UWS
P9	26	M	MVA	3	142	8	A1V1M3O2C0Ar1	MCS−
P10	22	F	MVA	3	1,900	5	A1V0M2O1C0Ar1	UWS

*The initial GCS (iGCS) is a range defined by the best (i.e., highest) and worst (i.e., lowest) post-resuscitation GCS scores assessed by a qualified clinician who performed a reliable examination (not confounded by sedation and/or paralytics) prior to ICU admission. LoC is assessed immediately prior to fMRI *via* behavioral evaluation with the CRS-R as coma, UWS, MCS−, or MCS+. The subscales for the CRS-R are auditory function (A), visual function (V), motor function (M), oromotor function (O), communication (C), and arousal (Ar)*.

*CRS-R, Coma Recovery Scale-Revised; F, female; fMRI, functional MRI; GCS, Glasgow Coma Scale; LoC, level of consciousness; M, male; MCS−, minimally conscious state without language; MCS+, minimally conscious state with language; MVA, motor vehicle accident; Ped, pedestrian; T, intubated, TBI, traumatic brain injury; UWS, unresponsive wakefulness syndrome*.

### Hand Squeezing

**Figure 2:**
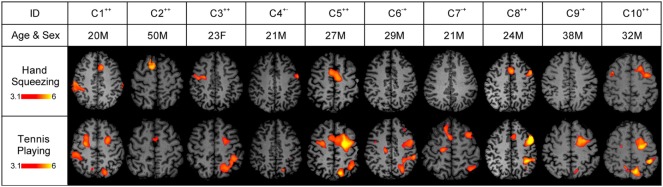
Stimulus-based functional magnetic resonance imaging (fMRI) responses to hand squeezing and tennis playing motor imagery paradigms in healthy subjects. fMRI data are shown as *Z*-statistic images to demonstrate stimulus-specific responses. *Z*-Statistic images are thresholded at cluster-corrected *Z* scores of 3.1 (inset color bars) and superimposed upon T1-weighted axial images. In the row-labeled “ID,” a “+” indicates that an fMRI response was detected and a “−” indicates that an fMRI response was not detected during the hand squeezing and tennis playing motor imagery paradigms, respectively. Abbreviations: F, female; M, male. All images are in radiologic convention.

**Figure 3:**
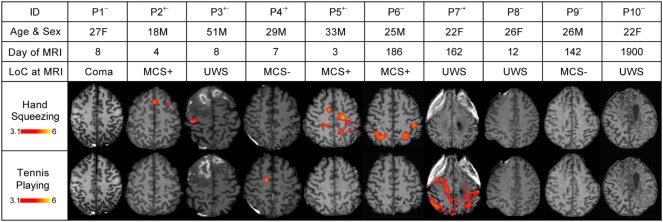
Stimulus-based functional magnetic resonance imaging (fMRI) responses to hand squeezing and tennis playing motor imagery paradigms in patients. fMRI data are shown as *Z*-statistic images to demonstrate stimulus-specific responses. *Z*-Statistic images are thresholded at cluster-corrected *Z* scores of 3.1 (inset color bars) and superimposed upon T1-weighted axial images. Level of consciousness (LoC) is assessed *via* behavioral evaluation with the Coma Recovery Scale-Revised as coma, unresponsive wakefulness syndrome (UWS), minimally conscious state without language (MCS−), or minimally conscious state with language (MCS+). In the row-labeled “ID,” a “+” indicates that an fMRI response was detected and a “−” indicates that an fMRI response was not detected during the hand squeezing and tennis playing motor imagery paradigms, respectively. Abbreviations: F, female; M, male. All images are in radiologic convention.

**Figure 4:**
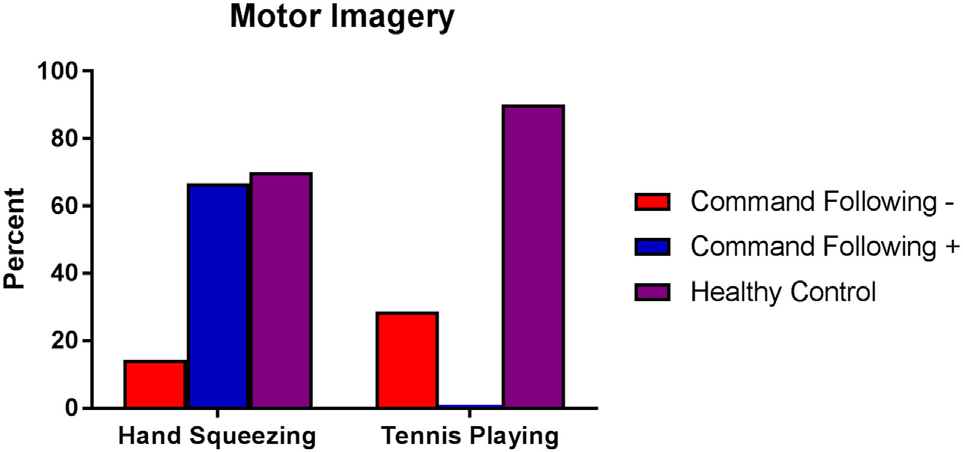
Percentage of healthy subjects, patients without behavioral evidence of command following [command following− (coma, UWS, MCS−)] and patients with behavioral evidence of command following [command following+ (MCS+)] who showed functional MRI responses to hand squeezing and tennis playing motor imagery tasks. Behavioral evaluation was performed using the Coma Recovery Scale-Revised. Healthy subjects are represented by purple bars, patients without behavioral evidence of command following by red bars, and patients with behavioral evidence of command following by blue bars. Abbreviations: UWS, unresponsive wakefulness syndrome; MCS−, minimally conscious state without language; MCS+, minimally conscious state with language; MRI, magnetic resonance imaging.

Seven of the 10 healthy subjects demonstrated covert command following *via* the hand squeeze paradigm [70%, 95% exact CI: (34.8–93.3%), see Table 2, Figures 2 and 4. Three of the 10 patients demonstrated command following on the hand squeeze paradigm [30% (6.7–65.3), see Table 3, Figures 3 and 4. Of the three patients with behavioral evidence of command following on bedside examination, two demonstrated command following on the hand squeezing fMRI task (TP = 2/3, FN = 1/3). Of the seven patients without behavioral evidence of command following, one demonstrated command following on the hand squeeze paradigm (TN = 6/7, FP = 1/7). Consequently, the sensitivity and specificity of the hand squeeze paradigm for detecting behavioral evidence of command following were 66.7% (12.5–98.2) and 85.7% (42.1–99.6), respectively, and ACC = 80%.

**Table 2:** Healthy control subject fMRI responses to hand squeeze and tennis motor imagery.

ID	Hand squeezing	Tennis playing
C1		+
C2	+	+
C3	+	+
C4	+	−
C5	+	+
C6	−	+
C7	−	+
C8	+	+
C9	−	+
C10	+	+
Total percent (95% exact CI)	70% (34.8–93.3)	90% (55.5–99.7)

*CI, confidence interval; fMRI, functional magnetic resonance imaging*.

**Table 3:** Patient fMRI responses to hand squeeze and tennis motor imagery.

ID	LoC at fMRI	Hand squeezing	Tennis playing
P1^a^	Coma	−	−
P2^a^	MCS+	+	−
P3^a^	UWS	+	−
P4^a^	MCS−	−	+
P5^a^	MCS+	+	−
P6	MCS+	−	−
P7	UWS	−	+
P8	UWS	−	−
P9	MCS−	−	−
P10	UWS	−	−
Total percent (95% exact CI)		30% (6.7–65.3)	20% (2.5–55.6)
Sensitivity (95% exact CI)		66.7% (12.5–98.2)	0% (0–70.8)
Specificity (95% exact CI)		85.7% (42.1–99.6)	71.4% (29.0–96.3)
Accuracy		80%	50%

*Sensitivity, specificity, and accuracy of motor imagery paradigm detection of command following in patients who demonstrated behavioral evidence of command following*.

*fMRI, functional magnetic resonance imaging; LoC, level of consciousness; MCS−, minimally conscious state without language; MCS+, minimally conscious state with language; UWS, unresponsive wakefulness syndrome; CI, confidence interval*.

*^a^The patient was receiving sedatives at the time of data acquisition (see Table S3 in Supplementary Material for details). Image acquisition parameters for P10 differed from those of the other subjects (see Materials and Methods for details)*.

### Tennis Playing

Nine of the 10 healthy subjects demonstrated command following *via* the tennis playing paradigm [90% (55.5–99.7)]. Two of the 10 patients demonstrated command following on the tennis playing paradigm [20% (2.5–55.6)]. Of the three patients with behavioral evidence of command following on bedside examination, none demonstrated command following on the tennis playing imagery paradigm (TP = 0/3, FN = 3/3). Of the seven patients without behavioral evidence of command following, two demonstrated command following on the tennis playing motor imagery paradigm (TN = 5/7, FP = 2/7). The sensitivity and specificity for the tennis playing paradigm for detecting behavioral evidence of command following were 0% (0–70.8) and 71.4% (29.0–96.3), respectively, and ACC = 50%.

The agreement between presence or absence of command following *via* hand squeezing and tennis playing imagery was 60% in healthy subjects and 50% in patients. Data on the percentage of voxels activated for each subject in each paradigm are presented in Tables S2 and S4 in Supplementary Material for healthy subjects and patients, respectively. For both the hand squeeze and tennis imagery paradigms, at least one healthy subject demonstrated 0% activated voxels. Therefore, all patients with >0% activated voxels in each paradigm met the predetermined criteria for having a positive response to the fMRI tasks (i.e., the ROI had >0% activated voxels and the percentage of activated voxels in the ROI exceeded the 2.5th percentile of the normal range in healthy subjects).

### Effect of Sedation on Behavioral Diagnosis and fMRI Responses

Administration of sedation (*n* = 5) was not associated with LoC or fMRI responses at the time of fMRI (Fisher’s Exact Test, df = 1; *P* = 0.17–0.99 for all analyses). The types and doses of sedative, anxiolytic, and analgesic medications administered at the time of fMRI are reported in Table S3 in Supplementary Material.

## Discussion

Precise assessment of LoC in patients with severe TBI is critical for guiding clinical management, providing accurate prognoses, and gaining access to rehabilitative services. Assessment of command following is a central component of the bedside examination and diagnostic impression. However, behavioral bedside assessment is susceptible to patient-, environment-, and examiner-related biases that contribute to the high misdiagnosis rate in this patient population. Multiple fMRI paradigms that probe covert consciousness have been investigated as potential objective markers of command following, but few studies have compared the utility of these paradigms concurrently in the same patient sample (31). Head-to-head comparison of fMRI command following tasks is needed to guide paradigm selection in future studies and eventually clinical practice. In addition, defining intrasubject variability would provide an objective basis for the cautious interpretation of absent fMRI responses to motor imagery paradigms. In this study, we assessed the sensitivity, specificity, and ACC of hand squeezing and tennis playing motor imagery paradigms in a sample of patients with severe TBI whose behavioral diagnosis at the time of the fMRI was coma, UWS, or MCS.

Although the tennis paradigm had higher ACC for detecting covert command following in healthy subjects, the hand squeezing paradigm accurately identified two of three patients who demonstrated behavioral evidence of command following (MCS+) as well as one patient (UWS) who did not. Conversely, responses to the tennis paradigm were absent in all three patients who demonstrated behavioral evidence of command following and present in two (MCS−, UWS) who did not. The sensitivity and sensitivity, specificity, and ACC of these findings suggest that the hand squeezing paradigm is a better classifier of command following in patients who are known to follow commands at the bedside than is the tennis playing paradigm.

One objective of developing fMRI paradigms for detection of conscious awareness in DoC is the prospect of identifying patients who retain the cognitive capacity for command following but do not demonstrate it at the bedside due to confounding factors such as impartments in speech or motor function. This cohort, described as having CMD (24, 26), is at risk for early withdrawal of life-sustaining therapies and denial of access to rehabilitative care. Our tennis playing paradigm identified two such patients, neither of whom showed fMRI responses to the hand squeezing paradigm, while the hand squeezing paradigm identified one such patient. For the purpose of this study, which focused on identifying the fMRI paradigm that best detects command following and therefore tried to maximize the TPR, these patients were included in the “false positive” group when calculating specificity and ACC. However, rather than the fMRI findings being “false positives,” it is possible that these three patients retained the cognitive capacity for command following but only demonstrated it on one of the two fMRI paradigms and not on bedside evaluation. Thus, the rate of detecting CMD was higher for the tennis playing paradigm than hand squeezing, even though the hand squeezing paradigm identified patients with behavioral evidence of command following with a higher ACC than did tennis. Future studies should address this apparent discrepancy between overall ACC and CMD detection rate by increasing the sample size, increasing the number of experimental runs, and interleaving the presentation of the fMRI paradigms. When possible, administering more than one type of command following paradigm should be considered to maximize the probability of detecting conscious awareness.

The tennis playing paradigm performed better than the hand squeezing task in the healthy control group while the opposite was true for patients. Comparison of fMRI activation profiles between healthy subjects and brain-injured patients must be performed with caution, given the multitude of factors that may influence an individual’s fMRI response. For example, in healthy subjects, playing tennis, whether on the court or in one’s imagination, may be a more cognitively challenging and salient task compared to a mundane hand-squeezing task. Conversely, hand squeezing may become more salient in a brain-injured patient who is asked to perform this task frequently during neurological examinations in the ICU. Furthermore, it is likely that imagining playing tennis requires multimodal processing and therefore would be expected to evoke a more distributed network than the unimodal task of imagining squeezing one’s hand. If so, patients, as compared to healthy subjects, may have access to less of the distributed network required to mediate tennis imagery due to focal lesions and loss of connectivity. For those patients who are able to cognitively perform the task, the frequent repetition of the command in the clinical environment may be associated with a mental training effect and/or an increased effort applied to the task and hence a more robust fMRI response (45). It is also possible that the patients who followed commands at the bedside were actually squeezing their hand in the scanner, rather than imagining the movement, leading to more robust SMA/PMC activation. In this study, we did not systematically record hand movements during the scanning session. Future studies that compare fMRI paradigms in this patient population should consider incorporating visual or electromyographic monitoring into their paradigm.

Several limitations should be considered when interpreting the results of this study. First, although this is one of the only studies that compares the ACC of fMRI motor imagery paradigms in a patient cohort, the sample size is small and includes patients in both acute and chronic phases of recovery. Consequently, generalizing our findings to other patient groups should be done with caution and future investigations should consider larger sample sizes of more homogenous patient groups enrolled across multiple sites. Second, the CIs around the fMRI command following proportions are very wide, suggesting that our sample has high variability and that the findings may lack precision when compared to the overall population of patients with traumatic DoC. Third, at the time of the fMRI scan, several subjects were receiving pharmacological sedation. Sedatives were administered at the discretion of the treating clinicians for patient safety or comfort and therefore could not be lifted for this study. Indeed, even patients who are comatose may require sedation to treat bronchospasm caused by an endotracheal tube. Sedation is therefore a medical necessity for some critically ill patients, and it is therefore unlikely that any fMRI study of critically ill patients that aims to be generalizable will be able to exclude patients receiving sedation.

Although the effects of sedating agents on cortical responses (46) and connectivity (47) are beginning to be elucidated, the impact of these pharmacological interventions may vary with multiple patient-specific factors, including tolerance, body mass, and metabolism. Furthermore, standardized sedation rating scales that have been validated in non-brain injured critically ill patients (e.g., the Richmond Agitation Sedation Scale) (48) are not applicable in severely brain-injured patients, for whom the behavioral effects of sedation cannot be quantitatively distinguished from the behavioral effects of the brain injury itself. As a result, we could not quantitatively measure the effect of sedation for each patient prior to scanning. Therefore, despite the lack of a statistical association between sedation and fMRI responsiveness, it is still possible that sedation affected the fMRI responses, though the results of the hand and tennis motor imagery paradigms would have been affected equally as all data were collected during the same scanning session.

Finally, we could not avoid some methodological challenges such as the hand squeezing motor imagery task always preceding the tennis playing motor imagery task. Though our findings do not suggest that this fixed order contributed to systematically poor arousal and therefore decreased fMRI responses to the tennis playing paradigm, future studies should consider randomizing the presentation of the tasks. In addition, imaging parameters for one patient, who did not show responses to either paradigm, did not conform to those of the other subjects, potentially adding variability to the data. The examiner completing the behavioral assessments for this study was not blinded to the previously determined clinical diagnosis of the patient (i.e., the diagnosis of LoC rendered by the treating physicians and nurses) and the investigator conducting the imaging analysis was not blinded to the behavioral assessment. Therefore, it is possible that the clinical diagnosis biased the behavioral assessment reported here and that, despite the automated nature of the imaging analysis, the behavioral assessment influenced the fMRI results. To avoid this potential limitation in the future, the behavioral assessment completed for study purposes should be conducted by an examiner who is not involved in the clinical care of the patient or in screening patients for inclusion in the study and the investigator conducting the imaging analysis should be blinded to the behavioral diagnosis. Finally, despite recent evidence supporting the need for serial behavioral assessment of LoC to improve diagnostic accuracy (16), we were only able to administer one CRS-R prior to the fMRI study due to the medical instability of the acutely ill patients and the limited time available to examine chronic patients returning for clinical follow-up. Future studies should aim to administer multiple behavioral assessments to establish the diagnostic baseline.

It is important to note that there was poor agreement between fMRI responses occurring during the hand squeezing and tennis playing fMRI squeezing tasks in both healthy subjects and patients. Specifically, all the healthy subjects who failed to demonstrate an fMRI response to hand squeezing or tennis imagery did show an fMRI response to the other paradigm. All of the patients who demonstrated an fMRI response to hand squeezing or tennis playing failed to show an fMRI response to the other paradigm. Fluctuation in arousal, task characteristics, prior exposure to the sport, and intersubject variability may have contributed to the inconsistencies in these findings. Future studies could consider individualizing the task such that it matches each participant’s experiences or interviewing participants and surrogates to ascertain prior exposure.

In choosing the appropriate task for detecting consciousness in patients diagnosed with DoC, investigators should carefully consider the research aims because a series of decisions (e.g., paradigm, analytic pipeline, and interpretation algorithm) related to the specific question of interest may alter the study design and findings. Furthermore, factors unrelated to cognitive ability, such as subject familiarity with the imagined task (e.g., a patient who has played tennis may be more responsive than a patient who has only watched the game on television) and analytic strategies (e.g., objective application of *a priori* ROIs versus subjective reading of fMRI activation maps) may introduce uncertainty into the data. Thus, different fMRI paradigms may lead to variable results in the same patient. We found that in patients with severe TBI diagnosed with DoC, the hand squeezing motor imagery paradigm detected covert command following with greater ACC than the tennis playing paradigm. However, the tennis playing paradigm was more sensitive in healthy subjects and identified more patients with CMD. These findings should be considered hypothesis-generating and will require replication in a larger sample of patients across multiple clinical and research sites. Clinical implementation of fMRI motor imagery paradigms for detection of consciousness will require further development, validation, and optimization of standardized approaches to fMRI data acquisition, analysis and interpretation.

## Ethics Statement

This study was carried out in accordance with a protocol approved by the Partners Institutional Review Board. Patient surrogate decision-makers gave written informed consent in accordance with the Declaration of Helsinki.

## Author Contributions

The authors contributed to the following aspects of the study: YB, JG, and BE: substantial contributions to the conception or design of the work; or the acquisition, analysis, or interpretation of data for the work; drafting the work or revising it critically for important intellectual content; final approval of the version to be published; and agreement to be accountable for all aspects of the work in ensuring that questions related to the accuracy or integrity of any part of the work are appropriately investigated and resolved.

## Conflict of Interest Statement

The authors declare that the research was conducted in the absence of any commercial or financial relationships that could be construed as a potential conflict of interest.

## Footnotes

^1^https://www.commondataelements.ninds.nih.gov.

^2^www.fmrib.ox.ac.uk/fsl.

## Abbreviations

DoC, disorders of consciousness; fMRI, functional magnetic resonance imaging; ICU, intensive care unit; LoC, level of consciousness; MCS±, minimally conscious state plus/minus; PTCS, post-traumatic confusional state; TBI, traumatic brain injury; UWS, unresponsive wakefulness syndrome.
