# Supplementary material for: Functional MRI Motor Imagery Tasks to Detect Command Following in Traumatic Disorders of Consciousness
Source: Front Neurol. 2017 Dec 18;8:688. doi: 10.3389/fneur.2017.00688 (PMC5741595; doi:10.3389/fneur.2017.00688)
Supplement: Supplementary file 1 [file Data_Sheet_1.docx]

**Supplementary Material**

**Functional MRI Motor Imagery Tasks to Detect**

**Command Following in Traumatic Disorders of Consciousness**

Bodien, YB^1,2^, Giacino, JT^2,3^ Edlow, BL^1,4^

^1^Center for Neurotechnology and Neurorecovery, and Laboratory for NeuroImaging of Coma and Consciousness, Department of Neurology, Massachusetts General Hospital and Harvard Medical School, Boston, MA, USA

^2^ Department of Physical Medicine and Rehabilitation, Spaulding Rehabilitation Hospital, Harvard Medical School, Boston, MA, USA

^3^Department of Psychiatry, Massachusetts General Hospital and Harvard Medical School, Boston, MA USA

^4^Athinoula A. Martinos Center for Biomedical Imaging, Massachusetts General Hospital and Harvard Medical School, Charlestown, MA, USA

*Correspondence:

Yelena G. Bodien, PhD

Center for Neurotechnology and Neurorecovery

Massachusetts General Hospital

175 Cambridge Street – Suite 300

Boston, MA 02114, USA

ybodien@mgh.harvard.edu

**Supplementary Table 1: Instructions given prior to and during functional MRI**

|  | **Hand Squeeze*** | **Tennis**** |
| --- | --- | --- |
| **Instructions**  **Prior to Stimulus** | When the MRI starts, close your eyes and relax. After about a minute, you will hear an instruction to squeeze your right hand. When you hear this, try to imagine that you are squeezing your right hand into a fist and then relaxing it. Concentrate on the way your muscles would feel if you were really performing this movement. Try to do this every time you hear the command to squeeze your right hand. Then, you will hear an instruction to stop. When you hear “stop”, stop squeezing your hand, relax, and keep your eyes closed. | When the MRI starts, close your eyes and relax. After about a minute, you will hear an instruction to play tennis. When you hear this, try to imagine you have a tennis racket in your hand and you are hitting the ball very hard back and forth with your opponent, as if you were on a tennis court during a competition. Keep imagining that you are playing tennis every time you hear the command to play tennis. Then you will hear an instruction to stop and rest. When you hear this, stop imagining that you are playing tennis, relax and keep your eyes closed. |
| **Instructions**  **During Stimulus** | At the beginning of the task, the subject hears “squeeze your right hand.” Then every 6 seconds the subject hears, “keep squeezing.” At the beginning of rest, the subject hears, “stop and rest.” Then every 6 sec, the subject hears “keep resting.” | At the beginning of the task, the subject hears “play tennis.” Then every 6 seconds the subject hears, “keep playing.” At the beginning of rest, the subject hears, “stop and rest.” Then every 6 sec, the subject hears “keep resting.” |

**Supplementary Table 1 Legend:** *The instructions for the hand squeeze motor imagery task were adapted from Cruse et al. Lancet 2011;378:2088-94. **The instructions for the tennis playing motor imagery task were adapted from Boly et al. Neuroimage 2007;36:979-92.

**Supplementary Table 2: Healthy subject responses to functional MRI motor imagery paradigms**

| ID | Age (years) | Sex | Hand Squeeze  (% activated voxels) | Tennis  (% activated voxels) |
| --- | --- | --- | --- | --- |
| C1 | 20 | M | 1.4% | 9.8% |
| C2 | 50 | M | 2.4% | 1.0% |
| C3 | 23 | F | 2.8% | 4.9% |
| C4 | 21 | M | 2.2% | 0.0% |
| C5 | 27 | M | 8.3% | 21.6% |
| C6 | 29 | M | 0.0% | 16.5% |
| C7 | 21 | M | 0.0% | 5.8% |
| C8 | 24 | M | 4.3% | 12.1% |
| C9 | 38 | M | 0.0% | 7.0% |
| C10 | 32 | M | 4.3% | 11.7% |
| Group Median | 28.5 | NA | 2.57% | 9.04% |
| Group Range | 21-50 | NA | 0-4.3% | 0-21.6% |

**Supplementary Table 2 Legend**: Abbreviations: F = female; fMRI = functional magnetic resonance imaging; M = male; NA = not applicable.

**Supplementary Table 3: Sedative, anxiolytic and analgesic medications administered before and during fMRI**

| **ID** | **LoC at**  **fMRI** | **Medications Administered**  **Before and During fMRI** |
| --- | --- | --- |
| P1 | Coma | Before MRI: propofol 50 mg/hr IV gtt  During MRI: propofol 50 mg/hr IV gtt |
| P2 | MCS+ | Before MRI: propofol 300 mg/hr IV gtt  During MRI: propofol 300 mg/hr IV gtt |
| P3 | UWS | Before MRI: fentanyl 50 mcg IV bolus + propofol 20 mg IV bolus  During MRI: propofol 25 mg/hr IV gtt |
| P4 | MCS- | Before MRI: propofol 300 mg/hr IV gtt + fentanyl 50 mcg IV bolus  During MRI: propofol 200 mg/hr IV gtt |
| P5 | MCS+ | Before MRI: propofol 300 mg/hr IV gtt + fentanyl 50 mcg IV bolus  During MRI: propofol 300 mg/hr IV gtt |
| P6 | MCS+ | None |
| P7 | UWS | None |
| P8 | UWS | None |
| P9 | MCS- | None |
| P10 | VS | None |

**Supplementary Table 3 Legend:** Level of Consciousness (LoC) was assessed via behavioural evaluation with the Coma Recovery Scale-Revised as coma, unresponsive wakefulness syndrome (UWS), minimally conscious state without language function (MCS-), or minimally conscious state with language function (MCS+). Abbreviations: fMRI = functional MRI; gtt = continuous infusion; IV = intravenous.

**Supplementary Table 4: Patient responses to functional MRI motor imagery paradigms**

| ID | LoC at  fMRI | Hand Squeeze  (% activated voxels) | Tennis  (% activated voxels) |
| --- | --- | --- | --- |
| P1 | Coma | 0.0% | 0.0% |
| P2 | MCS+ | 2.2% | 0.0% |
| P3 | UWS | 0.9% | 0.0% |
| P4 | MCS- | 0.0% | 1.3% |
| P5 | MCS+ | 7.5% | 0.0% |
| P6 | MCS+ | 0.0% | 0.0% |
| P7 | UWS | 0.0% | 0.5% |
| P8 | UWS | 0.0% | 0.0% |
| P9 | MCS- | 0.0% | 0.0% |
| P10 | UWS | 0.0% | 0.0% |
| Group Median | NA | 0% | 0% |
| Group Range | NA | 0.0-7.5% | 0.0-1.3% |

**Supplementary Table 4 Legend**: Level of Consciousness (LoC) is assessed via behavioural evaluation with the Coma Recovery Scale-Revised (CRS-R) as coma, unresponsive wakefulness syndrome (UWS), minimally conscious state without language function (MCS-), or minimally conscious state with language function (MCS+). Abbreviations: F = female; M = male; NA = not applicable.
